# Patients with uHCC and Child-Pugh B8/9 also benefit from a combination of antiangiogenic agents and PD-1 inhibitors: a multicenter real-world study

**DOI:** 10.2340/1651-226X.2025.42652

**Published:** 2025-05-05

**Authors:** Xiaoyan Ding, Xue Yin, Linlin Zheng, Lin Zhou, Junke Hu, Wei Sun, Lei Sun, Yanjun Shen, Ying Teng, Yawen Xu, Wendong Li, Mei Liu, Jinglong Chen

**Affiliations:** a Department of Cancer Center, Beijing Ditan Hospital, Capital Medical University, Beijing, China; b Jinan Eco-environmental Monitoring Center of Shandong Province, Jinan, Shandong Province, China; c Department of Interventional Radiology, The Fifth Medical Center, Chinese PLA General Hospital, Beijing, China; d Department of Pathology, Beijing Ditan Hospital, Capital Medical University, Beijing, China; e Department of Oncology, Beijing You’an Hospital, Capital Medical University, Beijing, China

**Keywords:** Hepatocellular carcinoma, lenvatinib, sorafenib, immune checkpoint inhibitors, child-pugh B

## Abstract

**Background and purpose:**

Patients with unresectable hepatocellular carcinoma (uHCC) and Child-Pugh grade B face limited treatment options and poor outcomes. This study aims to evaluate whether the effect and safety of combining tyrosine kinase inhibitors (TKIs) with progressive disease (PD)-1 inhibitors in uHCC patients with Child-Pugh B7 (CP7) and B8/9 (CP8/9) differ.

**Methods:**

This multicenter retrospective study included 179 uHCC patients with Child-Pugh B (CP7 group: *n* = 106; CP8/9 group: *n* = 73), receiving a combination of lenvatinib/sorafenib/other TKIs and PD-1 inhibitors between December 2020 and March 2023. Progression-free survival (PFS) and overall survival (OS) were defined as the primary endpoint. Secondary endpoints included the objective response rate (ORR) and safety.

**Results:**

The median PFS and OS for the entire cohort were 7.3 months (95% confidence intervals [CI]: 6.3–8.3) and 16.0 months (95% CI: 12.9–19.1), respectively. No statistically significant differences were observed between CP7 and CP8/9 groups in PFS (7.8 vs. 6.3 months, *p* = 0.28), OS (17.8 vs. 14.0 months, *p* = 0.20), ORR (33.0% vs. 27.4%, *p* = 0.42), or safety profiles. However, the CP8/9 group had significantly higher rates of TKI dose reductions (46.6% vs. 31.1%, *p* = 0.04) and discontinuations (57.5% vs. 24.5%, *p* < 0.001). Notably, 30.2% of patients maintained sustained radiographic responses despite advanced liver dysfunction.

**Interpretation:**

Combining TKIs with PD-1 inhibitors is an effective and well-tolerated option for HCC patients with Child-Pugh B, including those with CP8/9.

## Introduction

Hepatocellular carcinoma (HCC) accounts for 90% of primary liver tumors and is the third leading cause of cancer-related deaths worldwide [1, 2]. HCC develops after chronic liver injury and/or cirrhosis, which impacts treatment options. The severity of cirrhosis can be categorized into subgroups A, B, and C using the Child-Pugh score, which is based on laboratory values and clinical assessments. Because HCC often has an occult onset and atypical symptoms, 60% to 70% of cases are initially diagnosed as unresectable, and systemic therapy is recommended as the standard first-line regimen [2]. However, more than 70% of patients with unresectable hepatocellular carcinoma (uHCC) have concurrent cirrhosis, and up to 30% of these patients have grade B liver function [3–7]. Although many antiangiogenic tyrosine kinase inhibitors (TKIs) and immune checkpoint inhibitors (ICIs) have been used in clinical practice for uHCC since 2007, the median overall survival (mOS) has been relatively unchanged in patients with uHCC and Child-Pugh B (CP), ranging from 4.0 to 9.0 months [3–7]. In addition, given that patients with CP B have been excluded from large-scale, multicenter, phase III, randomized controlled clinical trials such as REFLECT [8], ORIENT-32 [9], and IMbrave 150, systemic treatment options for these patients remain limited [10].

To date, National Comprehensive Cancer Network (NCCN) guidelines have only recommended sorafenib (GIDEON) and nivolumab (CheckMate-040) for advanced HCC with CP B-graded liver function [3, 4, 7]. In a large retrospective study of 510 patients with advanced HCC and CP B liver function, the mOS was 4.0 months for sorafenib and 5.0 months for first-line nivolumab [5]. Lenvatinib is another first-line TKI that was studied in the phase III global multicenter REFELECT study. The lenvatinib arm showed significant improvements in median progression-free survival (mPFS) and objective response rate (ORR) compared to the sorafenib arm (mPFS 7.4 vs. 3.7 months; ORR 21.5% vs. 8.3%) [8]. Although no statistical difference was found in OS, in the subgroup analysis of Chinese patients, the mOS in the lenvatinib group was 15.0 months compared to 10.2 months in the sorafenib group [8]. In a meta-analysis, lenvatinib was shown to be significantly more effective than sorafenib in treating hepatitis B virus (HBV)-positive HCC patients (hazard ratios [HR] = 0.82) [11]. However, HCC patients with grade B liver function were not included in the REFLECT study [8]. In a retrospective study of 181 patients with advanced HCC who received lenvatinib as a first-line regimen (126 grade A; 55 grade B), the ORR in HCC with liver function grade A5 was significantly higher than that in HCC with grade A6 or grades B7 and 8 (44.0, 25.5, 22.2, and 5.3%, respectively [*p* = 0.002]) [6]. Another large-scale study enrolled 343 patients with advanced HCC, including 67 patients with CP B, and found a mOS of 9 months. The discontinuation rate was 70.1% in grade B compared with 69.2% in grade A [12]. uHCC patients with CP B liver functions continue to have low ORRs and poor prognoses, suggesting an urgent need to explore new treatment options.

Increasing numbers of preclinical studies and clinical reports have demonstrated synergy in angiogenesis by targeting Vascular Endothelial Growth Factor/Vascular Endothelial Growth Factor Receptor (VEGF/VEGFR) plus progressive disease (PD)-1/PD-L1 inhibitors [13, 14]. Combination regimens have been the primary and preferred choice for uHCC, and the combination of sorafenib plus PD-1 inhibitors or lenvatinib plus PD-1 inhibitors was administered to patients with uHCC and CP A liver function. However, it is not recommended in the guidelines for uHCC with CP B liver function, and data on dual regimens in uHCC with CP B are limited. Thus, we performed this multicenter retrospective study to evaluate the combination of TKIs and PD-1 inhibitors in patients with CP B. The purpose was to compare the effect and safety in patients with advanced liver dysfunction (CP8/9) with patient with CP7 and to determine whether these therapies provide a viable treatment alternative for patients with advanced liver dysfunction.

## Methods

### Study design and patients

This multicenter retrospective study was conducted at three separate centers in China (Capital Medical University Affiliated Beijing Ditan Hospital, The Fifth Medical Cancer, Chinese PLA General Hospital, and Capital Medical University Affiliated Beijing You’an Hospital). We included patients diagnosed with uHCC and CP B liver function between 31 December 2020 and 30 March 2023. According to CP scores, the enrolled patients were divided into two groups, CP B7 (CP7) and CP8/9.

The inclusion criteria were as follows: Patients should have ≥ 1 typically enhancing measurable target lesion ≥ 1 cm based on the modified Response Evaluation Criteria in Solid Tumors (mRECIST) [15], a CP score ≥ 7–9, were older than 18 years, histologically or clinically confirmed diagnosis of HCC [16], a life expectancy ≥ 3 months, an Eastern Cooperative Oncology Group (ECOG) performance status of 0 or 1, normal renal function, and no prior systemic therapy. Additional inclusion criteria included Barcelona Clinic Liver Cancer (BCLC) stage B/C disease and postoperative disease recurrence. Patients received at least one cycle of systemic therapy (one dose of PD-1 inhibitor and sorafenib or one dose of PD-1 inhibitor and lenvatinib). Patients with extrahepatic tumor spread or portal vein tumor thrombus (PVTT) were permitted except for those with symptomatic brain metastases. Patients were excluded if they had complete obstruction invasion of the primary branch of the biliary tract or had received any prior systemic therapy. In addition, patients were excluded if they had received any local treatment for HCC or radiotherapy for intrahepatic lesions for 4 weeks or three months, respectively, before giving informed consent. Patients with serious comorbidities, incomplete data, or who were lost to follow-up were also excluded. All patients included in the final analysis had complete baseline and outcome data.

This study was conducted in accordance with Good Clinical Practice, the principles outlined in the Declaration of Helsinki, and local laws. All participants provided a written informed consent before enrollment. This study has been approved by the Ethics Committee of the Capital Medical University affiliated Beijing Ditan Hospital (Ethical approval number: JDLKZ-2021-041-01).

### Treatments

Sorafenib (Bayer, Leverkusen, Germany, Sor) was initially administered orally at 200 mg twice daily. Lenvatinib (Lenvima^®^; Eisai Co., Ltd., Tokyo, Japan, Len) was administered orally daily according to body weight: Patients weighing < 60 kg received 4mg of lenvatinib once daily; patients weighing ≥ 60 kg received 8 mg of lenvatinib once daily.

Patients received 200 mg of sintilimab, camrelizumab, or tislelizumab intravenously on day 1 of a 21-day cycle following the first dose of sorafenib or lenvatinib. These agents were managed according to local regulations with dose reductions or treatment interruptions in the event of disease progression or unacceptable toxicity. Any drug interruptions and dose reductions were recorded, as well as the reasons for these events.

### Study endpoints

PFS and OS were designed as primary endpoint. Secondary endpoints included tumor response, ORR, and safety. The PFS was defined as the time from combination therapy commencement until disease progression according to mRECIST criteria or patient death, and the OS was defined as the time from the date of enrollment to death due to any cause. Tumor responses were assessed using contrast-enhanced dynamic computed tomography (CT) or magnetic resonance imaging (MRI) and by mRECIST. The best overall response was considered to be the final response. Final responses were documented as either complete response (CR), partial response (PR), stable disease (SD), or PD. The percentage of patients achieving a CR/PR was defined as ORR [15]. PVTT was considered a non-target lesion. In both groups, tumor responses to the entire treatment regimen were evaluated every 8–12 weeks after enrollment. Adverse events (AEs) were graded and recorded using the National Cancer Institute Common Terminology Criteria for Adverse Events version 4.18 [17].

### Statistical analyses

Statistical analysis was performed using R software (4.0.5). Demographic data and disease characteristics were compared between patients in the CP7 and CP8/9 group. Categorical variables were presented as numbers (percentages) and compared using Pearson’s χ^2^ test. Continuous variables were presented as mean ± standard deviation (SD) or median (interquartile range), with differences assessed using the T-test or Mann-Whitney U test, as appropriate. The ORR, disease control rate (DCR), and AEs were compared between the CP7 and CP8/9 groups using the χ^2^ test or Fisher’s exact test, depending on the data distribution. Survival curves for OS and PFS were estimated using the Kaplan-Meier estimator, and between-group differences (CP7 vs. CP8/9) were assessed by the log-rank test. Subgroup analyses were prespecified to assess the effect of CP score on the survival outcomes across clinically relevant subgroups, such as age, sex, Eastern Cooperative Oncology Group Performance Status (ECOG PS) score, hepatitis virus status, tumor number, tumor size, albumin-bilirubin (ALBI) grade, PVTT, extrahepatic metastasis, and treatment strategies. Within each subgroup, HRs and 95% confidence intervals (CIs) were calculated using proportional hazards models. All tests were two-sided, and a *p* < 0.05 was considered statistically significant.

## Results

### Patient baseline characteristics

**Figure 1:**
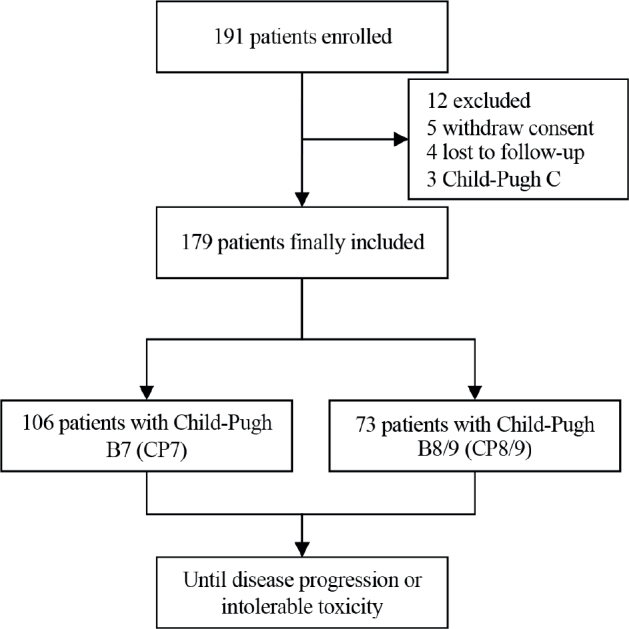
Study flowchart of the current study.

A total of 191 HCCs with CP B-graded liver function who were diagnosed between 31 December 2020 and 30 March 2023 at one of the three included hospital sites were initially recruited. Subsequently, 5 withdrew consent, 4 were lost to follow-up, and 3 had CP C liver function, leaving 179 patients included in the final analysis (Figure 1**)**. Based on their CP scores, patients were divided into two groups: CP7 (*n* = 106) and CP8/9 (*n* = 73). The median age was 61 (interquartile range: 53–68), with the majority being male (84.9%). Additionally, a total of 153 patients (85.5%) had HBV. Among them, 110 patients (61.5%) presented with types I-IV PVTT, and 47.5% (85 patients) of the cohort had extrahepatic metastasis. Regarding treatment regimens, 24.6% of patients received sorafenib plus PD-1 inhibitors, while 72.1% received lenvatinib plus PD-1 inhibitors. Prior treatments included hepatectomy (5.6%), transarterial chemoembolization (TACE) (20.7%), and ablation (15.6%). Furthermore, 163 patients (91.1%) had alpha-fetoprotein (AFP) levels ≥ 400 ng/mL. Moreover, 163 patients (91.1%) were classified as ALBI grade 1-2, with a significantly higher percentage in the CP7 group (98.1%) compared to the CP8/9 group (80.8%). In terms of ECOG PS, 88 patients (49.2%) had an ECOG score of 1, with a significantly higher proportion in the CP8/9 group (75.3%) than in the CP7 group (31.1%) (Table 1).

**Table 1:** Baseline patient demographic and disease characteristic.

Characteristics	CP7 (*n* = 106)	CP8/9 (*n* = 73)	*p*
Age (median, IQR) (61 [53–68])	59 [57–61]	62 [57–63]	0.70
Sex (Male/Female, 151/28)	93 (87.7%)/13(12.3%)	58 (79.5%)/15 (20.5%)	0.13
ECOG PS score (*n*, %)			<0.001
0 (91, 50.8%)	73 (68.9%)	18 (24.7%)	
1 (88, 49.2%)	33 (31.1%)	55 (75.3%)	
Hepatitis virus status (*n*, %)			0.25
HBV (153, 85.5%)	92 (86.8%)	61 (83.6%)	
HCV (17, 9.5%)	11 (10.4%)	6 (8.2%)	
Non-HBV or HCV (9, 5.0%)	3 (2.8%)	6 (8.2%)	
Group			0.87
SOR+PD-1 (44, 24.6%)	27 (25.5%)	17 (23.3%)	
LEN+PD-1 (129, 72.1%)	75 (70.8%)	54 (74.1%)	
Other+PD-1(6, 3.4%)	4 (3.8%)	2 (2.7%)	
Extrahepatic metastasis (*n*, %)			0.48
Yes (85, 47.5%)	58 (54.7%)	36 (49.3%)	
No (94, 52.5%)	48 (45.3%)	37 (50.7%)	
PVTT (*n*, %)			0.20
Yes (110, 61.5%)	61 (57.5%)	49 (67.1%)	
No (69, 38.5%)	45 (42.5%)	24 (32.9%)	
Tumor size (median, range)			0.24
(8.6 cm [6.9–10.8])	8.5 [8.3–9.7]	8.6 [7.6–9.4]	
Tumor number (*n*, %)			0.53
<3 (71, 39.7%)	40 (37.7%)	31 (42.5%)	
≥3 (108, 60.3%)	66 (62.3%)	42 (57.5%)	
AFP (ng/mL) (*n*, %)			0.06
≥400 ng/mL (163, 91.1%)	100 (94.3%)	63 (86.3%)	
<400 ng/mL (16, 8.9%)	6 (5.7%)	10 (13.7%)	
ALBI grade (*n*, %)			<0.001
1–2 (163, 91.1%)	104 (98.1%)	59 (80.8%)	
3 (16, 8.9%)	2 (1.9%)	14 (19.2%)	
BCLC			0.31
Stage B (28, 15.6%)	19 (17.9%)	9 (12.3%)	
Stage C (151, 84.4%)	87 (82.1%)	64 (87.7%)	
Tumor treatment history			
Hepatectomy (10, 5.6%)	6 (5.7%)	4 (5.5%)	0.96
TACE (37, 20.7%)	21 (19.8%)	16 (21.9%)	0.73
Ablation (28, 15.6%)	18 (17.0%)	10 (13.7%)	0.55

IQR: interquartile range; ECOG PS: Eastern Cooperative Oncology Group Performance Status; HBV: Hepatitis B virus; HCV: Hepatitis C virus; SOR: sorafenib; LEN, lenvatinib; PD-1: Programmed death receptor-1; PVTT: portal vein tumor thrombus; AFP: Alpha-fetoprotein; ALBI: Albumin-bilirubin; BCLC: Barcelona Clinic Liver Cancer, TACE: Transcatheter arterial chemoembolization.

### Survival analyses

**Figure 2:**
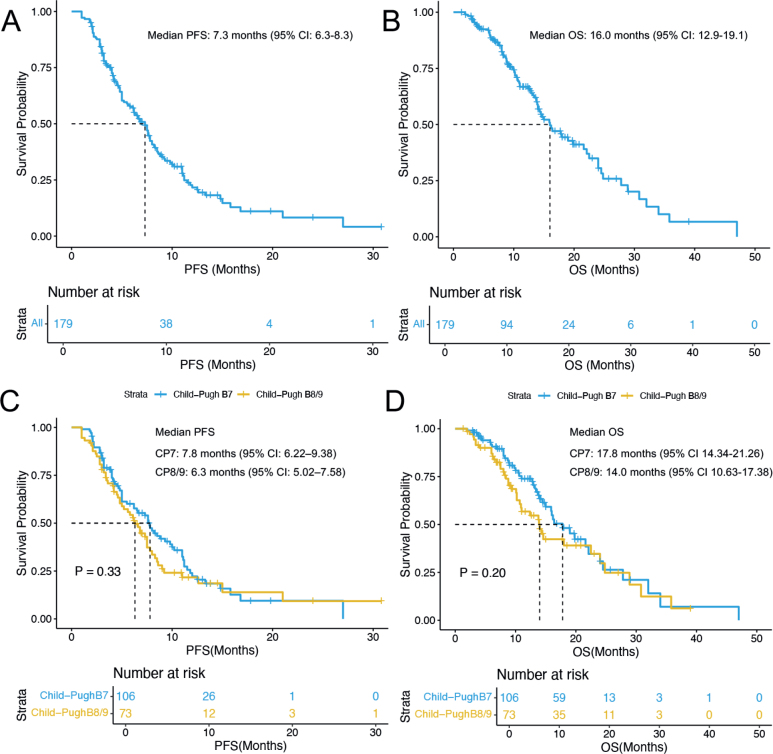
Kaplan–Meier curves for PFS and OS in the entire cohort (A, B) and in the Child-Pugh groups (C, D). PFS, Progression-free survival; OS, Overall survival.

The mPFS and mOS for the entire cohort were 7.3 months (95% CI: 6.3–8.3) and 16.0 months (95% CI: 12.9–19.1), respectively (Figure 2A and B). By the time of last follow-up, 84 patients had died, with 77 cases dying of disease progression. Among the remaining seven deaths, four were attributed to gastrointestinal hemorrhage (one in the CP7 group and three in the CP8/9 group), one patient in the CP8/9 group succumbed to liver failure, one in the CP7 group died from immune hemolytic anemia, and one in the CP8/9 group passed away due to Coronavirus Disease 2019 (COVID-19). Survival analysis between CP groups revealed comparable survival outcomes. The mPFS was 7.8 months (95% CI: 6.22–9.38) in the CP7 group and 6.3 months (95% CI: 5.02–7.58) in the CP8/9 group, with no statistically significant difference observed (*p* = 0.28; Figure 2C). Similarly, the mOS did not differ significantly between the two groups (17.8 months [95% CI: 14.34–21.26] vs. 14.0 months [95% CI: 10.63–17.38]; *p* = 0.20; Figure 2D).

### Subgroup analyses

**Figure 3:**
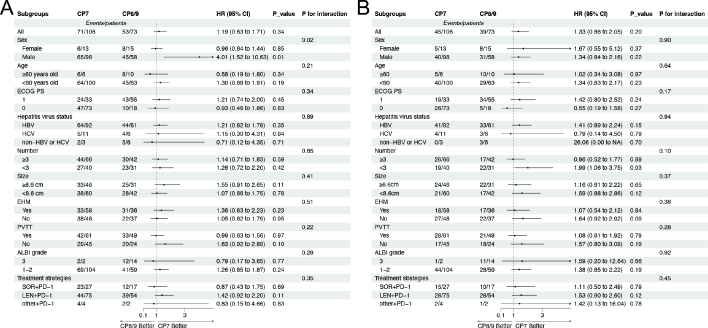
Forest plots of PFS (A) and OS (B) in patient subgroups. Each stratification was adjusted for age, sex, ECOG PS, etiology, tumor burden, ALBI grade, and treatment strategy. PFS, Progression-free survival; OS, Overall survival; ECOG PS, Eastern Cooperative Oncology Group Performance Status; HBV, Hepatitis B virus; HCV, Hepatitis C virus; EHM, Extrahepatic metastasis; PVTT, Portal vein tumor thrombus; ALBI, Albumin-bilirubin; SOR, Sorafenib; LEN, Lenvatinib; PD-1, Programmed death receptor-1.

The effect of CP score on survival outcomes was evaluated across clinically relevant subgroups. A significant interaction was detected between CP and sex for PFS (*p* for interaction = 0.02). Male patients in the CP7 group exhibited significantly longer PFS compared to those in the CP8/9 group (HR = 4.01, 95% CI: 1.52–10.63; *p* = 0.01), while no difference was observed in females (*p* = 0.85) **(** Figure 3A **)**. Notably, the proportion of male patients was higher in the CP7 group, which may have contributed to this observed difference (Table 1). Among patients with <3 tumors, those in the CP7 group showed longer OS compared to those in the CP8/9 group (HR = 1.99, 95% CI: 1.06–3.75; *p* = 0.03; Figure 3B). However, the interaction was not significant (*p* for interaction = 0.12), suggesting that this difference may be attributed to random variability rather than a true effect modification, or that the statistical precision was too low, due to the small sample sizes in the subgroups (*n* = 106 and *n* = 73). For the most part, survival outcomes remained consistent across other subgroups, suggesting that the PFS and OS for both CP7 and CP8/9 patients were generally comparable.

### Best response

**Figure 4:**
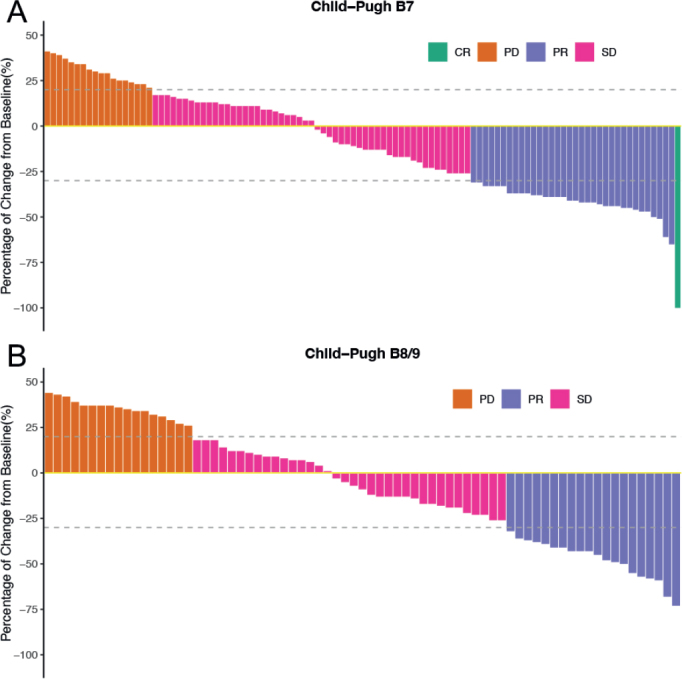
Best change from bassline in sum of longest target lesion diameters. (A) Best change in the Child-Pugh B7 group; (B) Best change in the Child-Pugh B8/9.

Best responses in the CP groups, as assessed by mRECIST criteria, are summarized in Table 2. A total of 126 patients developed disease progression till the last follow-up. Among the 106 patients in the CP7 group, 0.6% (*n* = 1) achieved a CR, 32.1% (*n* = 34) achieved a PR, 50.0% (*n* = 53) had SD, and 17.0% (*n* = 18) had PD (Figure 4A). In comparison, 27.4% (*n* = 20) of the 73 patients in the CP8/9 group had a PR, 49.3% (*n* = 36) had SD, and 23.3% (*n* = 17) had PD (Figure 4B**)**. With a median follow-up of 13.8 months, no statistically significant difference was found in the ORR (33.0% vs. 27.4%, *p* = 0.42) and DCR (83.0% vs. 76.7%, *p* = 0.30) between the CP7 and CP8/9 groups.

**Table 2:** Therapeutic response to sorafenib or lenvatinib in combination with PD-1 inhibitors for HCC with Child-Pugh B.

Efficacy	B7 (*n* = 106)	B8/9 (*n* = 73)	*p*
Complete response (*n*, %)	1 (0.6)	0	-
Partial response (*n*, %)	34 (32.1)	20 (27.4)	-
Stable disease (*n*, %)	53 (50.0)	36 (49.3)	-
Progression disease (*n*, %)	18 (17.0)	17 (23.3)	-
Objective response rate (*n*, %)	35 (33.0)	20 (27.4)	0.42
Disease control rate (*n*, %)	88 (83.0)	56 (76.7)	0.3

PD-1: Programmed death receptor-1; HCC: Hepatocellular carcinoma.

### Safety

Treatment-related AEs are presented in Table 3. In this study, the majority of patients (92.2%, *n* = 165) experienced AEs, with 45.8% (*n* = 82) reporting treatment-related AEs ≥ grade 3. In the CP8/9 group, the incidences of AEs of any grade and those of grade ≥ 3 were 93.2% and 49.3%, respectively. Safety profiles were comparable between the CP8/9 and CP7 groups. Common AEs in the CP8/9 group included hypertension, fatigue, and diarrhea, while rash, fatigue, and hypertension were common in the CP7 group. In addition, approximately 30.2% (*n* = 54) of patients developed immune-related adverse events (irAEs), with immune thrombocytopenia, hepatotoxicity, and hypothyroidism being the most common. About half of these patients required steroids. The incidence of gastrointestinal hemorrhage was 8.2% (*n*= 6) in the CP8/9 group and 7.5% (*n* = 8) in the CP7 group (*p* = 0.87). Reported dose reductions (31.1% vs. 46.6%; *p* = 0.04) or discontinuations (24.5% vs. 57.5%; *p* < 0.001) for TKIs plus PD-1 inhibitor significantly differed between groups. Although 46.9% (*n* = 84) of patients died at the time of the last follow-up, no deaths were associated with the combination sorafenib-PD-1 inhibitor or the lenvatinib-PD-1 treatments.

**Table 3:** Adverse events.

Adverse Events	B7 (*n* = 106)	B8/9 (*n* = 73)	*p*
Total treatment-emergent AEs	99 (93.4%)	68 (93.2%)	1
Total treatment-related treatment-emergent AEs	97 (91.5%)	68 (93.2%)	0.69
Treatment-emergent AEs (grade ≥3)	49 (46.2%)	37 (50.7%)	0.56
Treatment-related treatment-emergent AEs (grade ≥3)	46 (43.4%)	36 (49.3%)	0.44
Serious treatment-emergent AEs	12 (10GI Hemorrhage, 2ALT/AST elevation)	7 (6GI H, 1 immune hemolytic anemia)	
Serious treatment-related treatment-emergent AEs SAEs (grade 5)	11 (9GI Hemorrhage; 2ALT/AST elevation) 2 (2 GI Hemorrhage)	7 (6GI H, 1 immune hemolytic anemia) 2 (1GI H, 1 immune hemolytic anemia)	
IrAE	37 (34.9%)	17 (23.3%)	0.10
Grades 3–4	6* (5.7%)	1** (1.4%)	0.24
Grade 5	0	1***(1.4%)	0.41
Treatment-emergent adverse events occurring in ≥ 25% of patients in either group			
Anorexia			
Any grade	35 (33.0%)	24 (32.9%)	0.98
Grade ≥3	0	0	--
Increased blood bilirubin			
Any grade	40 (37.7%)	28 (38.4%)	0.93
Grade ≥3	11 (10.4%)	6 (8.2%)	0.63
Decreased serum albumin			
Any grade	36 (34%)	21 (28.8%)	0.46
Grade ≥3	6 (5.7%)	4 (5.5%)	1
Hypertension			
Any grade	48 (45.3%)	26 (35.6%)	0.20
Grade ≥3	6 (5.7%)	6 (8.2%)	0.55
Diarrhea			
Any grade	32 (30.2%)	29 (39.7%)	0.19
Grade ≥3	5 (4.7%)	4 (5.5%)	1
Ascites			
Any grade	40 (37.7%)	36 (49.3%)	0.12
Grade ≥3	5 (4.7%)	5 (6.8%)	0.74
ALT or AST elevation			
Any grade	41 (38.7%)	19 (26.0%)	0.08
Grade ≥3	4 (3.8%)	1 (1.4%)	0.65
Fatigue			
Any grade	33 (31.1%)	25 (34.2%)	0.66
Grade ≥3	2 (1.9%)	1 (1.4%)	1
Hypothyroidism			
Any grade	32 (30.2%)	18 (25.0%)	0.45
Grade ≥3	0	0	--
Hepatic encephalopathy			
Any grade	11 (10.4%)	6 (8.2%)	0.63
Grade ≥3	4 (3.8%)	2 (2.7%)	1
Dose reduction for TKIs	33 (31.1%)	34 (46.6%)	0.04
Proteinuria	7	3	
Diarrhea	3	3	
Fatigue	2	2	
Ascites	8	4	
Hepatic encephalopathy	8	4	
Grade 3 increased blood bilirubin	11	5	
Decreased platelet count	2	1	
Grade 4 ALT/AST elevation	2	0	
Grade 3 hypertension	4	1	
Discontinuation for TKIs	26 (24.5%)	42 (57.5%)	<0.001
Proteinuria	5	2	
Diarrhea	2	2	
Fatigue	1	3	
Ascites	6	3	
Hepatic encephalopathy	7	3	
Grade 3 increased blood bilirubin	9	3	
Hemorrhage, upper GI	11	6	
Grade 3 hypertension	2	0	
Anemia	0	1	
Decreased platelet count	2	0	

ALT: alanine aminotransferase; AST: aspartate aminotransferase; GI: gastrointestinal; AE: adverse events; IrAE: Immune-related adverse events; SOR: sorafenib; LEN: lenvatinib; TKIs: tyrosine kinase inhibitors.

* 1 case had hypophysitis, 2 case reported grade 3–4 ALT/AST elevation, 2 case had immune-related rash, and 1 case reported decreased platelet count.

** One case reported immune-related rash.

*** One case reported grade 5 immune hemolytic anemia.

## Discussion

Patients with CP8/9 are often excluded from clinical trials due to concerns about compromised hepatic reserve and limited treatment tolerance [8, 10, 18–20]. Consequently, their prognosis is determined not only by tumor burden but also by liver function [21, 22]. Previous studies have shown that untreated patients with CP B liver function have a median survival of 2–5 months, and monotherapies typically yield limited effect [23–25]. However, the potential benefits of combining TKIs with PD-1 inhibitors in CP B remain underexplored.

In this multicenter study, we evaluated the feasibility of the combination of sorafenib and lenvatinib-based TKIs and PD-1 inhibitors in patients with uHCC and CP B liver function. Our results revealed that patients in the CP8/9 group achieved a mPFS of 6.3 months and a mOS of 14.0 months, with an ORR of 27.4% and a DCR of 76.7%. These outcomes were comparable to those observed in the CP7 group, where the median PFS was 7.8 months, median OS was 17.8 months, ORR was 33.0%, and DCR was 83.0%. Notably, no significant differences were found between the two groups in terms of survival outcomes or safety profiles. Subgroup analyses further indicated that OS and PFS were consistent between CP7 and CP8/9 patients, regardless of the specific TKI and ICI combination regimen selection. Taken together, these results suggest that combination therapy is an effective and well-tolerated option for HCC patients with CP B, including those with CP8/9.

Our study underscores the survival benefits of the TKI and ICI combination therapy in CP B HCC patients. The mOS of 16.0 months and mPFS of 7.3 months across the entire cohort substantially surpass historical data for sorafenib or nivolumab monotherapy in CP B populations [3, 7, 26]. Monotherapy of TKI or ICI frequently encounters resistance due to tumor heterogeneity and compensatory signaling pathways [27–30]. The synergy between antiangiogenic agents and PD-1 inhibitors may overcome these limitations. Antiangiogenic agents normalize aberrant vasculature within tumors, thereby improving immune cell infiltration, while PD-1 blockade restores T-cell function, which is often suppressed by hypoxia-induced immunosuppression [9, 10, 31]. Although the immunosuppressive features of cirrhotic livers, ICIs can reverse this dysfunction by reprogramming tumor-associated macrophages and reinvigorating exhausted tumor-infiltrating lymphocytes [32, 33].

Our findings challenge the conventional exclusion of CP B8/9 patients from clinical trials, suggesting that the combination of TKI and PD-1 inhibitors may represent a viable therapeutic avenue in this population. Recent real-world studies have reported modest effect (ORR: 11–25%, median: PFS: 3.0–6.0 months, and mOS: 6.4–7.6 months) for the atezolizumab-bevacizumab combination in the CP B group [34–36]. The high predominance of HBV-related HCC (accounting for 85.5% of cases in our cohort) and the widespread use of lenvatinib (employed in 72.1% of combination therapies) may have contributed to enhanced responses, as prior studies have demonstrated the superior effect of lenvatinib in viral hepatitis-associated tumors [8, 11]. Moreover, the use of bevacizumab has been associated with risks of exacerbating portal hypertension, potentially contributing to the inferior survival outcomes seen in previous studies [10]. Our findings align with those studies that have explored the combination of camrelizumab and molecular targeted therapy (lenvatinib or sorafenib), which showed an ORR of 31.7%, median PFS of 5.1 months, and median OS of 13.4 months [37]. This further supports the effect of combining TKIs with ICIs for patients with advanced HCC, including those with CP B liver function.

Although the incidences of any grade of tumor and severe toxicity were higher in patients with CP B in our cohort, our findings were similar to those from other studies that have also included patients with CP B [2–4, 7, 26]. In our study, safety profiles were comparable between CP7 and CP8/9 patients, while the latter required more dose modifications (46.6% vs. 31.1%) and treatment discontinuations (57.5% vs. 24.5%). This may reflect the increased susceptibility of these patients to drug-related toxicities due to impaired hepatic function. Gastrointestinal hemorrhage was observed in 9.7% of cases, constituting the primary reason for treatment discontinuation, which was associated with the use of the sorafenib or lenvatinib. Nevertheless, no new safety signals emerged compared with prior clinical trials for sorafenib, lenvatinib, sintilimab, or camrelizumab [8, 9, 38, 39], and we found only one treatment-related death caused by irAE. Furthermore, another important safety concern is that patients with decompensated cirrhosis are more likely to have impaired liver function, upper gastrointestinal bleeding, and thrombocytopenia. Thus, liver function and symptoms of upper gastrointestinal bleeding should be monitored closely in this population.

Despite the promising results, our study has several limitations. First, the retrospective design may have introduced selection bias, and our cohort may not fully represent the broader HCC population. Moreover, the exclusion of patients with missing data relies on a missing completely at random assumption, which may not hold in practice. Future studies should implement advanced techniques, such as pattern-mixture models or machine learning-based imputation (e.g. MissForest with recursive feature elimination) to better address non-random missingness. Additionally, the use of multiple PD-1 inhibitors introduces heterogeneity in treatment effects, and we did not include a CP A control group for direct comparison of safety and efficacy. Nevertheless, our study is one of the few to include CP B8/9 patients, providing valuable real-world evidence of the potential benefit of combining TKIs and PD-1 inhibitors in this population.

## Conclusion

In conclusion, our study demonstrates that the combination of TKIs and PD-1 inhibitors offers a promising treatment option for HCC patients with CP B liver function, including those with CP8/9 scores. This combination therapy provides comparable PFS and OS outcomes between the CP7 and CP8/9 groups. Additionally, although the treatment is generally well tolerated, patients with CP8/9 may require closer monitoring due to an increased risk of treatment-related AEs.

### Statement of ethics

The study protocol, any amendments, and informed consent were approved by central or independent Institutional Review Board/Ethics Committee at participating sites (Approval number: JDLKZ-2021-041-01). This study was conducted in accordance with the principles of the Declaration of Helsinki, Good Clinical Practice guidelines, and local applicable regulatory requirements. All participants provided written-informed consent.

## Data Availability

The source data for this study are available from the corresponding author upon reasonable request.
